# *Journal of Veterinary Internal Medicine* Manuscript Reviewers Who Critiqued in the 2024 Calendar Year

**DOI:** 10.1111/jvim.70108

**Published:** 2025-05-02

The Editorial Board thanks the following individuals for their time and effort spent in reviewing and critiquing manuscripts during the 2024 calendar year.

Acierno, Mark—Arizona, United States

Adams, Larry—Indiana, United States

Adams, Paige—Oklahoma, United States

Adin, Christopher—Florida, United States

Adin, Darcy—Florida, United States

Adkins, Pamela—Missouri, United States

Afonso, Tiago—Saskatchewan, Canada

Aitee, Genna—Texas, United States

Aleman, Monica—California, United States

Allen, Justin—California, United States

Allenspach, Karin—Georgia, United States

Allerton, Fergus—England, United Kingdom of Great Britain and Northern Ireland

Almeida, Breno—São Paulo, Brazil

Alves, Lisa—United Kingdom of Great Britain and Northern Ireland

Aly, Sharif—California, United States

Ammersbach, Melanie—California, United States

Amorim, Irina—Portugal

Antonio, Bautista—Mexico

Apley, Michael—Kansas, United States

Appleman, Elizabeth—New York, United States

Arendt, Maja—Denmark

Armengou, Lara—Barcelona, Spain

Arnold, Susan—Minnesota, United States

Arostegui, Luis—Spain

Arroyo, Luis—Ontario, Canada

Asplin, John—Illinois, United States

Attipa, Charalampos—Merseyside, United Kingdom of Great Britain and Northern Ireland

Avery, Anne—Colorado, United States

Backus, Robert—Missouri, United States

Baka, Rania—Greece

Balkman, Cheryl—New York, United States

Bamford, Nicholas—Victoria, Australia

Bannister, Sidney—Florida, United States

Banse, Heidi—Arkansas, United States

Baral, Randolph—New South Wales, Australia

Barbosa da Costa, Guilherme—São Paulo, Brazil

Barge, Pablo—Switzerland

Barker, Lucy—United Kingdom of Great Britain and Northern Ireland

Barko, Patrick—Illinois, United States

Barnoon, Itai—United States

Baron Toaldo, Marco—Switzerland

Barr, Bonnie—Kentucky, United States

Barrett, Kirstie—California, United States

Barron, Lara—Hertfordshire, United Kingdom of Great Britain and Northern Ireland

Barrs, Vanessa—SAR China, Hong Kong

Basili, Mattia—Cheshire, United Kingdom of Great Britain and Northern Ireland

Bauquier, Jennifer—Victoria, Australia

Bayton, William—United Kingdom of Great Britain and Northern Ireland

Beccati, Francesca—United States

Beijerink, Niek—Netherlands

Benchekroun, Ghita—Île‐de‐France, France

Bennett, Peter—New South Wales, Australia

Berent, Allyson—New York, United States

Berghaus, Londa—Georgia, United States

Berghoff, Nora—Michigan, United States

Berman, Julie—Quebec, Canada

Bernier Gosselin, Véronique—Switzerland

Berryhill, Emily—California, United States

Bertin, Francois‐Rene—Indiana, United States

Bertram, Simon—United Kingdom of Great Britain and Northern Ireland

Bexfield, Nicholas—Cambridgeshire, United Kingdom of Great Britain and Northern Ireland

Bienzle, Dorothee—Ontario, Canada

Blackwood, Laura—Scotland, United Kingdom of Great Britain and Northern Ireland

Blais, Marie‐Claude—Quebec, Canada

Blois, Shauna—Ontario, Canada

Boag, Alisdair—United Kingdom of Great Britain and Northern Ireland

Bode, Elizabeth—Cheshire, United Kingdom of Great Britain and Northern Ireland

Boettcher, Irene—Germany

Bojanic, Krunoslav—Croatia

Bonagura, John—North Carolina, United States

Bonelli, Francesca—Italy

Bookbinder, Lauren—Michigan, United States

Bordin, Angela—Texas, United States

Borgeat, Kieran—United Kingdom of Great Britain and Northern Ireland

Bossens, Kenny—Belgium

Boswood, Adrian—Hertfordshire, United Kingdom of Great Britain and Northern Ireland

Botton, Sônia—Rio Grande do Sul, Brazil

Boudreau, C Elizabeth—Texas, United States

Boutet, Bruno—New Brunswick, Canada

Bracha, Shay—Ohio, United States

Brainard, Benjamin—Georgia, United States

Bray, Kathryn—United States

Bridglalsingh, Siobhan—Trinidad and Tobago

Bristow, Poppy—United Kingdom of Great Britain and Northern Ireland

Brockman, Daniel James—United States

Bröjer, Johan—Sweden

Buczinski, Sebastien—Quebec, Canada

Bullone, Michela—TO, Italy

Burchell, Richard—New Zealand

Burgess, Brandy—Georgia, United States

Burns, Teresa—Ohio, United States

Burton, Jenna—Colorado, United States

Busch, Rosie—California, United States

Busechian, Sara—Italy

Bush, William—Maryland, United States

Bussadori, Claudio—MI, Italy

Byrne, David—Western Australia, Australia

Byron, Julie—Ohio, United States

Cabrera Rocha, Catalina—Cyprus

Callahan, Trey—California, United States

Cameron, Starr—Wisconsin, United States

Cannon, Claire—Victoria, Australia

Capitan, Aurelien—France

Carr, Susan—Colorado, United States

Carvallo, Francisco—Virginia, United States

Casamian‐Sorrosal, Domingo—Spain

Catalfamo, Jim—New York, United States

Cauduro, Alberto—MI, Italy

Cavanaugh, Sarah—Saint Kitts and Nevis

Center, Sharon—New York, United States

Ceplecha, Vaclav—Czech Republic

Cerna, Petra—Colorado, United States

Cerón, José—Spain

Chako, Clemence—Arizona, United States

Chamorro, Manuel—Alabama, United States

Chan, Daniel—Hertfordshire, United Kingdom of Great Britain and Northern Ireland

Chan, Jennifer—California, United States

Chawla, Pranav—Ohio, United States

Chew, Dennis—Ohio, United States

Chon, Esther—Wisconsin, United States

Chow, Betty—California, United States

Christopherson, Peter—Alabama, United States

Churchill, Julie—Minnesota, United States

Cianciolo, Rachel—Ohio, United States

Clarke, Dana—Pennsylvania, United States

Clergue, Suzanne—France

Clifford, Craig—Pennsylvania, United States

Cloquell Miro, Ana—Glasgow, United Kingdom of Great Britain and Northern Ireland

Coates, Joan—Missouri, United States

Cogan, Tristan—United Kingdom of Great Britain and Northern Ireland

Coggins, Sally—New South Wales, Australia

Cole, Stephen—Pennsylvania, United States

Collings, Alison—United Kingdom of Great Britain and Northern Ireland

Collins, Niamh—New South Wales, Australia

Conley, Alan—California, United States

Constable, Peter—Illinois, United States

Contreras, Elena—New York, United States

Contreras, G Andres—Michigan, United States

Cook, Audrey—Texas, United States

Cooke, Kirsten—Florida, United States

Cooper, Edward—Ohio, United States

Corbee, Ronald—Netherlands

Cornelis, Ine—Belgium

Cosford, Kevin—Saskatchewan, Canada

Costa, Lais—California, United States

Costa, Marcio—Quebec, Canada

Cote, Etienne—Prince Edward Island, Canada

Couetil, Laurent—Indiana, United States

Cowgill, Larry—California, United States

Credille, Brenton—Georgia, United States

Cribb, Alastair—Massachusetts, United States

Cridge, Harry—Michigan, United States

Cullen, John—North Carolina, United States

Culp, William—California, United States

Cuq, Benoît—Ireland

Curran, Kaitlin—Oregon, United States

Daminet, Sylvie—Belgium

Dandrieux, Julien—Scotland, United Kingdom of Great Britain and Northern Ireland

Daniels, Joshua (Josh)—Colorado, United States

De Decker, Steven—Hertfordshire, United Kingdom of Great Britain and Northern Ireland

de Freitas, Maria Ines—United Kingdom of Great Britain and Northern Ireland

de Laat, Melody—Queensland, Australia

De Majo, Massimo—Italy

de Oliveira Conrado, Francisco—Massachusetts, United States

de Stefani, Alberta—Hertfordshire, United Kingdom of Great Britain and Northern Ireland

Dear, Jonathan—California, United States

DeDonder, Keith—Maryland, United States

Defarges, Alice—Ontario, Canada

DeFrancesco, Teresa—North Carolina, United States

DEL BALDO, FRANCESCA—Bologna, Italy

Del Nero, Bruna—Florida, United States

Dembek, Katarzyna—North Carolina, United States

Dennis, Ruth—Cambridgeshire, United Kingdom of Great Britain and Northern Ireland

Dennler, Matthias—Switzerland

Depenbrock, Sarah—California, United States

Devriendt, Nausikaa—Belgium

Dickerson, Vanna—Texas, United States

Dickson, Dave—United Kingdom of Great Britain and Northern Ireland

Distl, Ottmar—Germany

Divers, Thomas—New York, United States

Dixon, Claire—Massachusetts, United States

Djani, Dylan—South Carolina, United States

Dondi, Francesco—Bologna, Italy

Donnelly, Callum—New York, United States

Dore, Vincent—Quebec, Canada

Dorsch, Roswitha—Germany

dos Santos, Luis—Indiana, United States

Driver, Colin—United Kingdom of Great Britain and Northern Ireland

Dröes, Floris—Texas, United States

Duarte, Ricardo—São Paulo, Brazil

Dunkel, Bettina—Herts, United Kingdom of Great Britain and Northern Ireland

Durham, Andy—Hampshire, United Kingdom of Great Britain and Northern Ireland

Durward‐Akhurst, Sian—Minnesota, United States

Dutrow, Emily—New Jersey, United States

Duz, Marco—United Kingdom of Great Britain and Northern Ireland

Dye, Janice—United States

Eberhardt, Christina—South Africa

Egenvall, Agneta—Sweden

ElAshmawy, Wagdy—California, United States

El‐Hage, Charles—Victoria, Australia

Elliott, Jonathan—United Kingdom of Great Britain and Northern Ireland

El‐Tholoth, Mohamed—Dakahlia, Egypt

Epstein, Steven—California, United States

Ericsson, Aaron—Missouri, United States

Erkılıc, Ekin—Turkey

Espinosa, Pablo—Ontario, Canada

Estell, Krista—Virginia, United States

Eucares Von Laer, Anna—Brazil

Fadda, Angela—Avon, United Kingdom of Great Britain and Northern Ireland

Fajt, Virginia—Texas, United States

Faller, Kiterie—United Kingdom of Great Britain and Northern Ireland

Fan, Timothy—Illinois, United States

Fecteau, Marie‐Eve—Pennsylvania, United States

Feijoó, Silvia—Argentina

Fellman, Claire—Massachusetts, United States

Fenner, William—Ohio, United States

Ferlini Agne, Gustavo—South Australia, Australia

Ferrari, Roberta—Italy

Ferraro, Salvatore—Uppsala, Sweden

Fidel, Janean—Washington, United States

Finstad, Joanna—Ohio, United States

Fischer, Andrea—Bayern, Germany

Floyd, Emily—United Kingdom of Great Britain and Northern Ireland

Foss, Kari—Illinois, United States

Foster, Derek—North Carolina, United States

Foster, Jonathan (JD)—District of Columbia, United States

Fowlie, Sam—Essex, United Kingdom of Great Britain and Northern Ireland

Fox, Philip—New York, United States

Foy, Daniel—Arizona, United States

Fracassi, Federico—Italy

Freeman, Paul—United Kingdom of Great Britain and Northern Ireland

Freiche, Valerie—France

Frey, Erin—North Carolina, United States

Friedrichs, Kristen—Wisconsin, United States

Fries, Ryan—Illinois, United States

Furr, Martin—Oklahoma, United States

Furrow, Eva—Minnesota, United States

Gagnon, Allison—California, United States

Galac, Sara—Netherlands

Galati, Pamela—Mississippi, United States

Galvao, Felipe—Illinois, United States

Gamsjäger, Lisa—Alberta, Canada

Garabed, Rebecca—Ohio, United States

Garcia, Gabriel—Florida, United States

Garner, Bridget—Georgia, United States

Gaschen, Frederic—Louisiana, United States

Gaschen, Lorrie—Louisiana, United States

Geddes, Rebecca—United Kingdom of Great Britain and Northern Ireland

Gentile, Jessica—Maine, United States

German, Alexander—Cheshire, United Kingdom of Great Britain and Northern Ireland

Ghantous, Seth—Florida, United States

Gibbons, Philippa—Texas, United States

Gibbs, Nicole—North Carolina, United States

Gieger, Tracy—North Carolina, United States

Gilor, Chen—Florida, United States

Gin, Taylor—North Carolina, United States

Glaus, Tony—Switzerland

Goggs, Robert—New York, United States

Gold, Jenifer—Wisconsin, United States

Golini, Lorenzo—Zürich, Switzerland

Gomart, Samantha—United Kingdom of Great Britain and Northern Ireland

Gomes, Viviani—Sao Paulo, Brazil

Gomez, Diego—Ontario, Canada

Goncalves, Rita—United Kingdom of Great Britain and Northern Ireland

Goncalves, Ron—Florida, United States

Gookin, Jody—North Carolina, United States

Gorden, Patrick—Iowa, United States

Gordon, Daniel—Colorado, United States

Gostelow, Ruth—Hertfordshire, United Kingdom of Great Britain and Northern Ireland

Gould, Emily—Texas, United States

Graham, Joanne—Missouri, United States

Granger, L Abbigail—Louisiana, United States

Granger, Nicolas—United Kingdom of Great Britain and Northern Ireland

Granick, Jennifer—Minnesota, United States

Grant, David—Virginia, United States

Grenager, Nora—Virginia, United States

Grissett, Gretchen—Mississippi, United States

Grobman, Megan—Alabama, United States

Guglielmini, Carlo—Italy

Gull, Tamara—Missouri, United States

Gunn‐Moore, Danielle—United Kingdom of Great Britain and Northern Ireland

Gustafson, Daniel—Colorado, United States

Gutierrez‐Quintana, Rodrigo—United Kingdom of Great Britain and Northern Ireland

Hackett, Eileen—New York, United States

Hahn, Caroline—Scotland, United Kingdom of Great Britain and Northern Ireland

Halleran, Jennifer—North Carolina, United States

Hannabuss, Joshua—London, United Kingdom of Great Britain and Northern Ireland

Hansen, Bernie—North Carolina, United States

Harcourt‐Brown, Thomas (Tom)—Avon, United Kingdom of Great Britain and Northern Ireland

Hardefeldt, Laura—Victoria, Australia

Harper, Cindy—South Africa

Harris, Autumn—North Carolina, United States

Hart, Kelsey—Georgia United States

Hasegawa, Daisuke—Tokyo, Japan

Haspeslagh, Maarten—Belgium

Havel, Peter—California, United States

Hecht, Silke—Tennessee, United States

Hedgespeth, Barry—North Carolina, United States

Heilmann, Romy—Saxony, Germany

Heinze, Cailin—Kansas, United States

Hendricks, Jeanette—California, United States

Hepworth‐Warren, Kate—North Carolina, United States

Her, Jiwoong—Ohio, United States

Herstad, Kristin—Netherlands

Hess, Rebecka—Pennsylvania, United States

Hewetson, Michael—Hertfordshire, United Kingdom of Great Britain and Northern Ireland

Hezzell, Melanie—United Kingdom of Great Britain and Northern Ireland

Hill, Richard—Florida, United States

Hill, Tracy—Minnesota, United States

Hobbs, Kallie—Texas, United States

Hoenig, Margarethe—Illinois, United States

Hogan, Daniel—Indiana, United States

Hokamp, Jessica—Texas, United States

Holt, Natalee—Maine, United States

Hopster‐Iversen, Charlotte—Denmark

House, John—New South Wales, Australia

Hovda, Lynn—Minnesota, United States

Hsieh, Emmelyn—Pennsylvania, United States

Hsue, Weihow—New York, United States

Hulsebosch, Sean—California, United States

Hülsmeyer, Velia—Germany

Hume, Kelly—New York, United States

Humm, Karen—United Kingdom of Great Britain and Northern Ireland

Hunter, Robert—Kansas, United States

Husnik, Roman—Tennessee, United States

Igarashi, Hirotaka—Kanagawa, Japan

Intile, Joanne—North Carolina, United States

Ivester, Kathleen—Indiana, United States

Jablinski, Anne—Iowa, United States

Jablonski, Sara—Michigan, United States

Jackson, Wendi—California, United States

Jacob, Megan—North Carolina, United States

Jaffey, Jared—Arizona, United States

Jager, Mason—New York, United States

James, Fiona—Ontario, Canada

Jamieson, Camilla—Indiana, United States

Jaramillo, Camilo—California, United States

Jeffery, Nicholas—Texas, United States

Jepson, Rosanne—Herts, United Kingdom of Great Britain and Northern Ireland

Jergens, Albert—Iowa, United States

Jessen, Lisbeth—Denmark

Johnson, Amy—Pennsylvania, United States

Johnson, Lynelle—California, United States

Johnson, Philip—Missouri, United States

Johnson, Susan—Ohio, United States

Johnston, Andrea—Louisiana, United States

Jokar, Mohammad—Tehran, Iran (the Islamic Republic of)

Jokinen, Tarja—Finland

Jones, Gareth—United Kingdom of Great Britain and Northern Ireland

Jugan, Maria—Kansas, United States

Kadotani, Saki—Illinois, United States

Kalbfleisch, Theodore—Kentucky, United States

Kanazono, Shinichi—Saitama, Japan

Kang, Byeong‐Teck—Korea (the Republic of)

Kass, Philip—California, United States

Kathrani, Aarti—United Kingdom of Great Britain and Northern Ireland

Keebaugh, Audrey—Virginia, United States

Keene, Bruce—North Carolina, United States

Kelley, Ashley—Florida, United States

Kellihan, Heidi—Wisconsin, United States

Kelly, Darren—Hampshire, United Kingdom of Great Britain and Northern Ireland

Kendall, Allison—North Carolina, United States

Kent, Andrew—United Kingdom of Great Britain and Northern Ireland

Kidd, Linda—California, United States

King, Ailbhe—North Carolina, United States

KIRBAŞ, Akın—Turkey

Kirschvink, Nathalie—Belgium

Kittleson, Mark—California, United States

Knebel, Anna—Germany

Knipe, Marguerite—California, United States

Knowles, Edward—United States

Kohn, Barbara—Germany

Kook, Peter—Zurich, Switzerland

Kopecny, Lucy—California, United States

Kopper, Jamie—Washington, United States

Korman, Rachel—Queensland, Australia

Kornya, Matthew—Ontario, Canada

Kortum, Andre—Cambridgeshire, United Kingdom of Great Britain and Northern Ireland

Kraemer, Anna Lena—United Kingdom of Great Britain and Northern Ireland

Krainer, Dorothee—Ireland

Krause, Alexandre—Rio Grande do Sul, Brazil

Kretsch, Cileah—California, United States

Kreuder, Amanda—Iowa, United States

Kromhout, Kaatje—Belgium

KuKanich, Butch—Kansas, United States

KuKanich, Kate—Kansas, United States

Kurogochi, Kentaro—North Carolina, United States

Kurtz, Maxime—France

Kwiatkowska, Miloslawa—Poland

Labato, Mary Anna—Massachusetts, United States

Lacorcia, Lauren—United Kingdom of Great Britain and Northern Ireland

Ladlow, Jane—United Kingdom of Great Britain and Northern Ireland

Laflamme, Dorothy (Dottie)—Virginia, United States

Lai, Serene—Ontario, Canada

Lakritz, Jeff—Ohio, United States

Lale, Dilara—Switzerland

Landolt, Gabriele—Colorado, United States

Langlois, Daniel—Michigan, United States

Lappalainen, Anu—Finland

Lascelles, Duncan—North Carolina, United States

Lashnits, Erin—North Carolina, United States

Lathan, Patty—Louisiana, United States

Lawson, Jack—United Kingdom of Great Britain and Northern Ireland

Leal, Rodolfo—Portugal

Leclere, Mathilde—Quebec, Canada

Lee, Jih‐Jong—Taiwan

Lee, Robert—United States

Lee, Ya‐Jane—Taiwan

Leeb, Tosso—Switzerland

Leguillette, Renaud—Alberta, Canada

Lehmann, Hendrik—Austria

Leisewitz, Andrew—Alabama, United States

Lennon, Elizabeth—Pennsylvania United States

Lenz, Jennifer—Pennsylvania, United States

Letko, Anna—Switzerland

Lewis, Melissa—North Carolina, United States

Li, Ronald—North Carolina, United States

Liatis, Theofanis (Fanis)—United Kingdom of Great Britain and Northern Ireland

Lidbury, Jonathan—Texas, United States

Lilliehöök, Inger—Sweden

Lindåse, Sanna—Sweden

Lindley, Stephanie—Alabama, United States

Lisciandro, Gregory—Texas, United States

Ljungvall, Ingrid—Sweden

Lluesma, Begona—United Kingdom of Great Britain and Northern Ireland

Loftus, John—New York, United States

Long, Sam—Victoria, Australia

Lopez, Ignacio—Spain

Lourenço, Bianca—Georgia, United States

Lovett, Amy—New Zealand

Lubas, George—Italy

Luby, Christopher—Saskatchewan, Canada

Lucidi, Cynthia—Michigan, United States

Luethy, Daniela—Pennsylvania, United States

Lulich, Jody—Minnesota, United States

Lynch, Alex—North Carolina, United States

Lyu, Yang—East flanders, Belgium

Mabry, Kasey—Georgia, United States

Mackin, Andrew—Mississippi, United States

Mahony Wages, Orla—Massachusetts, United States

Malik, Richard—New South Wales, Australia

Malone, Erin—New York, United States

Manchester, Alison—Colorado, United States

Mandigers, Paul—Netherlands

Mansfield, Caroline—Victoria, Australia

Marioni‐Henry, Katia—United Kingdom of Great Britain and Northern Ireland

Marks, Stanley—California, United States

Marolf, Angela—Ohio, United States

Matiasek, Kaspar—Bavaria, Germany

Matsuyama, Arata—Ontario, Canada

Mauk, Michael—Pennsylvania, United States

May, Anna—Germany

Mayhew, Joe—New Zealand

Mazaki‐Tovi, Michal—Israel

Mazan, Melissa—Massachusetts, United States

Mcaloon, Conor—United States

McCleary‐Wheeler, Angela—Missouri, United States

McElroy, Abigail—Massachusetts, United States

McGovern, Kate—Illinois, United States

McKeever, Kenneth—New Jersey, United States

McKinney, Caroline—Massachusetts, United States

McVey, Alistair—Minnesota, United States

Meade, Kieran—Ireland

Melián‐Limiñana, Carlos—Las Palmas, Spain

Menciotti, Giulio—Virginia, United States

Menzies‐Gow, Nicola—Herts, United Kingdom of Great Britain and Northern Ireland

Meurens, Francois—France

Meyer, James—New York, United States

Meyerhoff, Nina—Lower Saxony, Germany

Mitchell, Katharyn—New York, United States

Mizuno, Takuya—Japan

Mochel, Jonathan—Georgia, United States

Moise, N Sydney—New York, United States

Monforte Monteiro, Susana—United Kingdom of Great Britain and Northern Ireland

Mooney, Carmel—Ireland

Moore, George—Indiana, United States

Moore, Sarah—Florida, United States

Moraiti, Katerina—Greece

Moreira da Silva, Joana—Portugal

Morey, Nekessa—United Kingdom of Great Britain and Northern Ireland

Morgan, Jessica—California, United States

Morris, Celeste—Missouri, United States

Morrison, Philippa—State/Province, United Kingdom of Great Britain and Northern Ireland

Morrow, Jennifer—Kentucky, United States

Motta, Jean‐Paul—France

Muñana, Karen—North Carolina, United States

Muñoz, Ana—Cordoba, Spain

Murakami, Keiko—Iowa, United States

Musteata, Mihai—Romania

Musulin, Sarah—North Carolina, United States

Mylonakis, Mathios—Greece

Nath, Laura—South Australia, Australia

Navarro‐Cubas, Xavier—United Kingdom of Great Britain and Northern Ireland

Naylor, Jonathan—Saskatchewan, Canada

Nemec Svete, Alenka—Slovenia

Nessler, Jasmin—Germany

Niedzwiedz, Artur—Poland

Niessen, Stijn—Herts, United Kingdom of Great Britain and Northern Ireland

Nishida, Hidetaka—Japan

Nissen, Sarah—Denmark

Noble, Glenys—New South Wales, Australia

Nolen‐Walston, Rose—Pennsylvania, United States

Norton, Elaine—Arizona, United States

Nostell, Katarina—Sweden

Nout‐Lomas, Yvette—Colorado, United States

Ohad, Dan—Jerusalem, Israel

Ojeda, Javier—Chile

Olby, Natasha—North Carolina, United States

Ollivett, Theresa—Wisconsin, United States

Oman, Rachel—Arizona, United States

O'Neill, Emma—Dublin, Ireland

Orvalho, Joao—California, United States

Pakozdy, Akos—Austria

Palerme, Jean‐Sébastien—Iowa, United States

Palm, Carrie—California, United States

Palus, Viktor—Slovakia

Papich, Mark—North Carolina, United States

Pariaut, Romain—New York, United States

Parker, Valerie—Ohio, United States

Parmentier, Thomas—Quebec, Canada

Partington, Catheryn—Cheshire, United Kingdom of Great Britain and Northern Ireland

Passler, Thomas—Alabama, United States

Patterson, Carly—Texas, United States

Patterson, Edward (Ned)—Minnesota, United States

Paul, Linda—South Carolina, United States

Pearl, David—Ontario, Canada

Pelligand, Ludovic—United Kingdom of Great Britain and Northern Ireland

Penning, Louis—Utrecht, Netherlands

Peralta, Santiago—New York, United States

Pereira, Nuno—ZH, Switzerland

Pesato, Michael—Delaware, United States

Peters, Rosanne—Illinois, United States

Petersen, Jessica—Nebraska, United States

Peterson, Mark—New York, United States

Physick‐Sheard, Peter—Ontario, Canada

Piercy, Richard—United Kingdom of Great Britain and Northern Ireland

Pinn‐Woodcock, Toby—New York, United States

Piotrowski, Isabelle—Switzerland

Poirier, Valerie—Ontario, Canada

Pollard, Rachel—California, United States

Pomrantz, Jill—Maine, United States

Potter, Brianna—Colorado, United States

Priestnall, Simon—Hatfield, United Kingdom of Great  Britain and Northern Ireland

Pritchard, Jessica—Wisconsin, United States

Pusterla, Nicola—California, United States

Quimby, Jessica—Ohio, United States

Raabis, Sarah—Colorado, United States

Raheb, Shari—Ontario, Canada

Raidal, Sharanne—New South Wales, Australia

Raimondi, Francesca—Hampshire, United Kingdom of Great Britain and Northern Ireland

Rajamäki, Minna—Finland

Ramey, Dave—Connecticut, United States

Ramsauer, Anna Sophie—Switzerland

Ramsey, Ian—United Kingdom of Great Britain and Northern Ireland

Rayhel, Laura—Arizona, United States

Reagan, Krystle—California, United States

Rebhun, Robert—California, United States

Reed, Stephen—Kentucky, United States

Reinhart, Jennifer—Illinois, United States

Reppert, Emily—Kansas, United States

Reuss, Sarah—United States

Rhinehart, Jaylyn—Ohio, United States

Richard, Eric—France

Ridyard, Alison—United Kingdom of Great Britain and Northern Ireland

Riese, Franziska—Germany

Riggers, Denise S—Germany

Rishniw, Mark—New York, United States

Rivas, Victor—North Carolina, United States

Rivero, Luis—Missouri, United States

Roberts, Veronica—North Somerset, United Kingdom of Great Britain and Northern Ireland

Robertson, Elise—United Kingdom of Great Britain and Northern Ireland

Rocha, Izabela—Kentucky, United States

Rohrer Bley, Carla—Switzerland

Rolph, Kerry—Saint Kitts and Nevis

Romito, Giovanni—Bologna, Italy

Rosenblatt, Alana—Australia

Rossi, Gabriele—Western Australia, Australia

Rossow, Heidi—California, United States

Rostoll‐Cangiano, Lautoro—Wisconsin, United States

Roura, Xavier—Spain

Rout, Emily—Colorado, United States

Royaux, Emilie—United Kingdom of Great Britain and Northern Ireland

Rozanski, Elizabeth—Massachusetts, United States

Rubio, Camila Peres—Spain

Ruby, Rebecca—Kentucky, United States

Rudinsky, Adam—Ohio, United States

Rusbridge, Clare—Surrey, United Kingdom of Great Britain and Northern Ireland

Rush, John—Massachusetts, United States

Rylander, Helena—Wisconsin, United States

Saito, Miyoko—Kanagawa, Japan

Sakamoto, Kaori—Georgia, United States

Saker, Korinn—North Carolina, United States

Salavati Schmitz, Silke—United Kingdom of Great Britain and Northern Ireland

Sanderson, Sherry—Georgia, United States

Sanz, Macarena—Iowa, United States

Saunders, Ashley—Texas, United States

Scansen, Brian—Colorado, United States

Schaefer, Deanna—Tennessee, United States

Schaefer, Emily—Virginia, United States

Schermerhorn, Thomas (Tom)—United States

Schober, Karsten—Ohio, United States

Schoeman, Johan—Gauteng, South Africa

Schott II, Harold—Michigan, United States

Schroeder, Eric—Ohio, United States

Schulz, Bianka—Germany

Schwarzwald, Colin—ZH, Switzerland

Scott, Katherine—United States

Scott‐Moncrieff, Catharine—Indiana, United States

Scudder, Christopher—Hertfordshire, United Kingdom of Great Britain and Northern Ireland

Segev, Gilad—Israel

Sellon, Rance—Washington, United States

Selting, Kim—Illinois, United States

Seo, Joonbum (Joon)—New Zealand

Serrano, Goncalo—Belgium

Seth, Mayank—United Kingdom of Great Britain and Northern Ireland

Shamir, Merav—Israel

Shane, Douglas—Kansas, United States

Sharkey, Leslie—Massachusetts, United States

Sharp, Claire—Western Australia, Australia

Sheahan, Breanna—North Carolina, United States

Sherding, Robert—Ohio, United States

Shnaiderman‐Torban, Anat—Israel

Shoveller, Anna Kate—Ontario, Canada

Shropshire, Sarah—Colorado, United States

Sieber‐Ruckstuhl, Nadja—Switzerland

Siegers, Esther—Netherlands

Silvestrini, Paolo—Pennsylvania, United States

Simpson, Kenneth—New York, United States

Siwinska, Natalia—Poland

Skelly, Barbara—Cambs, United Kingdom of Great Britain and Northern Ireland

Skinner, Stephanie—District of Columbia, United States

Slead, Tanner—North Carolina, United States

Smets, Pascale—Belgium

Smith, Fauna—California, United States

Smith, Joseph—Tennessee, United States

Smrkovski, Olya—Tennessee, United States

Snyder, Jessica—Washington, United States

Sojka Kritchevsky, Janice—Indiana, United States

Soltero‐Rivera, Maria—California, United States

Spada, Eva—Italy

Spalla, Ilaria—United Kingdom of Great Britain and Northern Ireland

Spugnini, Enrico—Italy

Stabile, Fabio—United Kingdom of Great Britain and Northern Ireland

Stalin, Catherine—United Kingdom of Great Britain and Northern Ireland

Staudacher, Anne—United Kingdom of Great Britain and Northern Ireland

Steffen, Frank—Zürich, Switzerland

Steinman, Amir—Israel

Stephenson, Hannah—Lancashire, United Kingdom of Great Britain and Northern Ireland

Stepien, Rebecca—Wisconsin, United States

Stevenson, Mark—Victoria, Australia

Stockman, Jonathan—New York, United States

Stoner, Caitlin—Ohio, United States

Stowe, Devorah—North Carolina, United States

Strohmeyer, Jessica—Michigan, United States

Suchodolski, Jan—Texas, United States

Sullivan, Edmund—Washington, United States

Sullivan, Stacey—Alabama, United States

Summers, Stacie—Oregon, United States

Sutherland, Brian—Georgia, United States

Suzuki, Ryohei—Tokyo, Japan

Swann, James—New York, United States

Swartz, Turner—United States

Syme, Harriet—Herts, United Kingdom of Great Britain and Northern Ireland

Syrja, Pernilla—Finland

Tallon, Rose—United Kingdom of Great Britain and Northern Ireland

Tamamoto, Takashi—Japan

Tamborini, Alice—Ireland

Tasker, Séverine—United Kingdom of Great Britain and Northern Ireland

Tate, Nicole—Minnesota, United States

Taxis, Tasia—Michigan, United States

Tayler, Sarah—Hertfordshire, United Kingdom of Great Britain and Northern Ireland

Taylor, Samantha—United Kingdom of Great Britain and Northern Ireland

Taylor‐Brown, Frances—United Kingdom of Great Britain and Northern Ireland

ter Woort, Fe—Belgium

Teske, Erik—Netherlands

Tessman, Ronald—Kansas, United States

Thomason, John—Mississippi, United States

Thomovsky, Elizabeth—Indiana, United States

Tidholm, Anna—Sweden

Tjostheim, Sonja—Wisconsin, United States

Toborowsky, Carl—Illinois, United States

Tolbert, M Katherine—Texas, United States

Toribio, Ramiro—Ohio, United States

Torres‐Henderson, Camille—Colorado, United States

Trepanier, Lauren—Wisconsin, United States

Troxel, Mark—Massachusetts, United States

Trub, Sarah—Virginia, United States

Tsamouri, Maria Malvina—California, United States

Turley, Kelsey—Ohio, United States

Turunen, Soile—Finland

Ueda, Yu—North Carolina, United States

Ullal, Tarini—California, United States

Valldecabres, Ainhoa—Ireland

van den Boom, Robin—Netherlands

van Eps, Andrew—Pennsylvania, United States

Van Erck‐Westergren, Emmanuelle—Belgium

Van Vertloo, Laura—Iowa, United States

Vandevelde, Marc—Switzerland

Vanhaesebrouck, An—United Kingdom of Great Britain and Northern Ireland

Vaughn, Sarah—Georgia, United States

Vezzosi, Tommaso—Pisa, Italy

Viall, Austin—California, United States

Viitanen, Sanna—Finland

Visser, Lance—Colorado, United States

Viviano, Katrina—Wisconsin, United States

von Euler, Henrik—Sweden

Walker, Julie—Wisconsin, United States

Walmsley, Gemma—United Kingdom of Great Britain and Northern Ireland

Ward, Jessica—Iowa, United States

Warland, James—Essex, United Kingdom of Great Britain and Northern Ireland

Warnken, Tobias—Lower Saxony, Germany

Washburn, Kevin—Texas, United States

Watson, Penny—United Kingdom of Great Britain and Northern Ireland

Weaver, Leslie—Kansas, United States

Webb, Craig—Colorado, United States

Webster, Cynthia—Massachusetts, United States

Weese, J (Scott)—Ontario, Canada

Wehner‐Fleischberger, Astrid—Germany

Weiblen, Carla—Brazil

Westropp, Jodi—California, United States

White, Julianne—Mississippi, United States

White, Stephen—California, United States

Whitehouse, William—Indiana, United States

Wilkins, Pamela—Illinois, United States

Willcox, Jennifer—California, United States

Willi, Barbara—Switzerland

Williams Louie, Elizabeth—New York, United States

Williams, David—Illinois, United States

Williams, Diane—California, United States

Windsor, Rebecca—Massachusetts, United States

Winston, Jenessa—Ohio, United States

Wismer, Tina—New York, United States

Witkowski, Lucjan—Poland

Wittenburg, Luke—California, United States

Witzel, Angela—Tennessee, United States

Woldemeskel, Moges—Georgia, United States

Wolf, Jacob—Florida, United States

Wolff, Ewan—Florida, United States

Wood, Carrie—Massachusetts, United States

Wood, Michael—Wisconsin, United States

Woods, Glynn—United Kingdom of Great Britain and Northern Ireland

Woods, J Paul—Ontario, Canada

Woolcock, Andrew—Indiana, United States

Wyatt, Sophie—Hertfordshire, United Kingdom of Great Britain and Northern Ireland

Xenoulis, Panagiotis—Texas, United States

Xifra Rubio, Pilar—Madrid, Spain

Yamasaki, Masahiro—Iwate, Japan

Yancey, Caroline—New York, United States

Yang, Vicky—Massachusetts, United States

Yanke, Amy—Alabama, United States

Young, Lesley—Suffolk, United Kingdom of Great Britain and Northern Ireland

Yu, Jane—New South Wales, Australia

Zakia, Luiza—Ontario, Canada

Zatelli, Andrea—Bari, Italy

Ziegler, Jessie—Arizona, United States

